# Effect of Dietary Linoleic Acid Intake on Eicosapentaenoic Acid Status and Lipoxygenase-Mediated Oxylipin Biosynthesis in Healthy Adults: A Randomized Controlled Trial

**DOI:** 10.3390/nu18111814

**Published:** 2026-06-04

**Authors:** Susan Sergeant, Linda H. Easter, Tammy Mustin, Priscilla Ivester, Jimaree A. Legins, Michael C. Seeds, Carrie S. Standage-Beier, Anderson Cox, Cristina M. Furdui, Brian Hallmark, Floyd H. Chilton

**Affiliations:** 1 Department of Biochemistry, Wake Forest School of Medicine, Medical Center Blvd, Winston-Salem, NC 27157, USA; susan.sergeant@wfusm.edu (S.S.); jimlegins@gmail.com (J.A.L.); 2 Office of Clinical and Academic Research, Wake Forest School of Medicine, Medical Center Blvd, Winston-Salem, NC 27157, USA; 3 Wake Forest Institute of Regenerative Medicine, Wake Forest School of Medicine, Medical Center Blvd, Winston-Salem, NC 27157, USA; michael.seeds@advocatehealth.org; 4 School of Nutritional Sciences and Wellness, University of Arizona, Tucson, AZ 85719, USA; cstandagebeir@arizona.edu; 5 Proteomics and Metabolomics Shared Resource, Comprehensive Cancer Center, Wake Forest School of Medicine, Medical Center Blvd, Winston-Salem, NC 27157, USA; anderson.cox@advocatehealth.org; 6 Department of Internal Medicine, Section on Molecular Medicine, Wake Forest School of Medicine, Medical Center Blvd, Winston-Salem, NC 27157, USA; cristina.furdui@advocatehealth.org; 7 BIO5 Institute, University of Arizona, 1657 E. Helen St., Tucson, AZ 85719, USA; bhallmar@arizona.edu; 8 Escuela de Medicina de la Salud, Tecnologica de Monterrey, Monterrey 64719, Mexico

**Keywords:** linoleic acid, eicosapentaenoic acid, oxylipins, dietary intervention, inflammation

## Abstract

Background/Objectives. The modern Western diet (MWD) provides high linoleic acid (LA) exposure, typically contributing 6–9% of the total caloric intake. These high LA levels have fueled a longstanding debate about whether this dietary pattern confers benefit or risk. Importantly, LA intake is disproportionately elevated among lower socioeconomic populations due to greater reliance on industrial seed oils and ultra-processed foods. Despite decades of research, controlled dietary intervention studies directly evaluating the biological consequences of varying LA exposure remain limited. Methods. The current randomized, double-blind intervention (ClinicalTrials.gov; NCT02962128; 11 November 2016) compared the effects of a 12-week Low-LA diet (2.5% energy) versus a High-LA diet (10.0% energy) in healthy adults. Outcomes included plasma concentrations of highly unsaturated fatty acids (HUFAs) and ex vivo zymosan-stimulated whole-blood oxylipin generation. Results. Fifty-two participants completed the intervention. High LA exposure resulted in marked reductions in plasma *n*-3 eicosapentaenoic acid (EPA) and eicosatetraenoic acid (ETA) concentrations compared with the Low-LA arm. Docosapentaenoic acid (DPA) was also significantly lower in weeks 4 and 8. In contrast, levels of the *n*-6 HUFA arachidonic acid (ARA) did not differ with dietary LA exposure. Conclusions. HUFA and oxylipin analyses revealed that higher dietary LA markedly increased the ratios of ARA to EPA and ARA- to EPA-derived oxylipin species, shifting the lipid mediator balance toward a more *n*-6-dominant inflammatory profile.

## 1. Introduction

Over the past century, shifts in agricultural practices toward the industrial production of commodity crops, particularly soybeans and corn, have transformed the global food supply and sparked a scientific and public health debate [1,2,3,4]. Central to this controversy is whether the widespread dietary use of end products derived from these crops, including linoleic acid (LA)-rich vegetable oils, is healthy. Increased dietary LA has contributed to a pervasive imbalance in dietary *n*-6 relative to *n*-3 polyunsaturated fatty acids (PUFA) that has been proposed to affect the incidence and progression of chronic diseases [5,6,7]. This issue has expanded globally with the adoption of the Modern Western Diet (MWD) which is characterized by a high intake of LA [8]. LA now constitutes a major component of both industrial food production and household cooking due to its low cost, long shelf life, and broad availability. Given their low cost, high prevalence in ultra-processed foods [9], extensive use in fast-food outlets, and limited access to healthy *n*-3-containing foods, the shift toward high-LA commodity seed oils has disproportionately affected lower-income populations [10,11,12].

Early guidance to increase dietary LA stemmed from the diet–heart hypothesis of the 1960s–1970s, which proposed that replacing saturated fats with LA-rich vegetable oils would lower serum cholesterol, thereby reducing the risk of atherosclerosis and coronary heart disease. Consistent with this framework, early prospective randomized controlled trials and longitudinal dietary interventions were designed to increase LA intake, primarily by substituting saturated fatty acids with vegetable oils [13,14,15,16,17]. However, initial reports from these randomized dietary trials were largely silent on the impact of LA-enriched interventions on other endpoints, such as cardiovascular and all-cause mortality. A re-analysis of the Sydney Diet Heart Study showed that replacing saturated fat with high-LA safflower oil markedly increased all-cause and cardiovascular mortality despite lowering cholesterol [18]. A related re-analysis of the Minnesota Coronary Experiment found no mortality benefit from lowering cholesterol via high-LA corn oil, and a signal toward higher mortality in some subgroups [19]. Finally, a 2010 meta-analysis by Ramsden et al. distinguished between mixed *n*-3/*n*-6 polyunsaturated fatty acid (PUFA) trials that showed reduced coronary heart disease (CHD) risk and *n*-6–specific PUFA trials (primarily LA), which showed no benefit and possible harm [20]. Importantly, the meta-analysis did not claim definitive harm across all contexts; rather, it showed that benefits previously attributed to *n*-6 PUFA were largely driven by trials that increased *n*-3 alongside *n*-6.

Although typically present at lower dietary abundance, *n*-3 highly unsaturated fatty acids (HUFA), especially eicosapentaenoic acid (EPA), have been shown to play critical roles in regulating inflammation, thrombosis, and immune responses by being converted into bioactive lipid mediators [21,22,23]. In contrast, metabolites of the *n*-6 HUFA, arachidonic acid (ARA), typically exhibit opposing biological activities, including promotion of inflammatory and pro-thrombotic signaling pathways [24,25]. Importantly, both EPA and ARA are synthesized via a common enzymatic pathway that includes precursor elongation, desaturation, membrane incorporation, and ultimately downstream HUFA oxylipin biosynthesis.

Competition between *n*-6 and *n*-3 PUFAs along the elongation–desaturation pathway was first demonstrated in rat liver microsomal systems [26,27] and in subsequent whole-animal feeding studies [28]. These studies confirmed that increasing dietary LA suppresses the conversion of alpha-linolenic acid (ALA) to EPA and docosahexaenoic acid (DHA) while reciprocally enhancing ARA synthesis from LA. Stable-isotope tracer and controlled feeding studies in humans demonstrate that higher dietary LA intake reduces the conversion of ALA to long-chain *n*-3 HUFAs, particularly EPA [29,30,31].

Because LA and ALA compete for the same elongation and desaturation enzymes, variation in dietary LA intake has been shown in in vitro and vivo animal models to shift metabolic flux through this shared pathway, thereby altering membrane HUFA composition. Yet human data across physiologically relevant ranges of LA intake remain limited. We therefore conducted a controlled clinical trial comparing High-LA and Low-LA dietary exposures to directly quantify how altering LA intake influences *n*-6 and *n*-3 HUFA synthesis and the subsequent production of bioactive lipid mediators derived from EPA and ARA. This design enables an important assessment of how habitual LA intake modulates the biochemistry underlying inflammatory and thrombotic signaling in humans.

## 2. Materials and Methods

**Figure 1:**
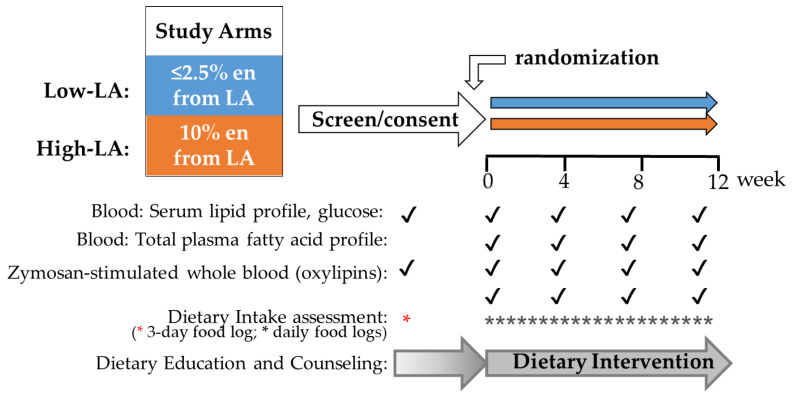
Study Design. The dietary intervention study used a double-blind, randomized, parallel-arm design. After consenting and completing a rigorous screening process, eligible participants were randomized to one of two dietary interventions and consumed either a Low-LA (2.5% EN) or High-LA (10% EN) diet, with constant ALA exposure in both arms for 12 weeks. Participants met with Registered Dietitians (RDs) weekly to pick up study foods, review weekly food logs, and receive ongoing dietary counseling. Fasting biospecimens and anthropometric and cardiovascular measurements were collected at 4-week intervals.

This dietary intervention study was designed to examine the effects of manipulating dietary LA exposure on plasma PUFA and HUFA levels and on zymosan whole blood-generated oxylipin levels in healthy participants consuming a controlled diet with either a Low (2.5% energy) or High (10% energy) LA environment for 12 weeks (Figure 1). Naturally occurring safflower variants producing seed oils with low (~13%) and high (~75%) LA contents were used to manipulate dietary LA exposure. ALA exposure (~1% energy) from flaxseed oil was kept consistent across both arms. The target for dietary fat calories was 30% for both arms. A double-blind, randomized, parallel, two-arm dietary intervention study design was employed. Approval from the Wake Forest University Health Sciences Institutional Review Board and the NIH/Office of Clinical Research Affairs was obtained before beginning the study, which was conducted in a clinical research setting with free-living participants living in and around Winston-Salem, NC, USA. The trial was registered with ClinicalTrials.gov (NCT02962128; registered 11 November 2016).

### 2.1. Study Participants

The study was conducted from August 2016 to December 2021 at Wake Forest University Health Sciences (WFUHS). The inclusion criteria were healthy adults of European or African ancestry (self-identifying as non-Hispanic), aged 21–65 years, and free of major diseases (as described below). Exclusion criteria included the following: (a) current use of anti-inflammatory drugs (NSAIDs, oral/IV steroids, other injectable anti-inflammatory drugs, >100 mg aspirin per day, leukotriene receptor antagonists), niacin, fibrates, or fish or botanical oils (containing PUFAs); (b) blood pressure > 140/90 mm Hg; (c) fasting blood triglycerides > 150 mg/dL and fasting blood glucose > 125 mg/dL; (d) pacemaker, defibrillator, myocardial infarction, vascular surgery, or stroke in the preceding year; (e) any stage heart failure, prior cholecystectomy, or end-stage renal disease; (f) BMI < 19 or >30; (g) pregnancy or nursing; (h) alcohol use >14 drinks per week; (i) self-reported current use of tobacco, marijuana, cannabidiol (CBD), or other illicit drugs; and (j) intolerance or allergy to safflower or flax oils or any component in the provided study foods.

**Figure 2:**
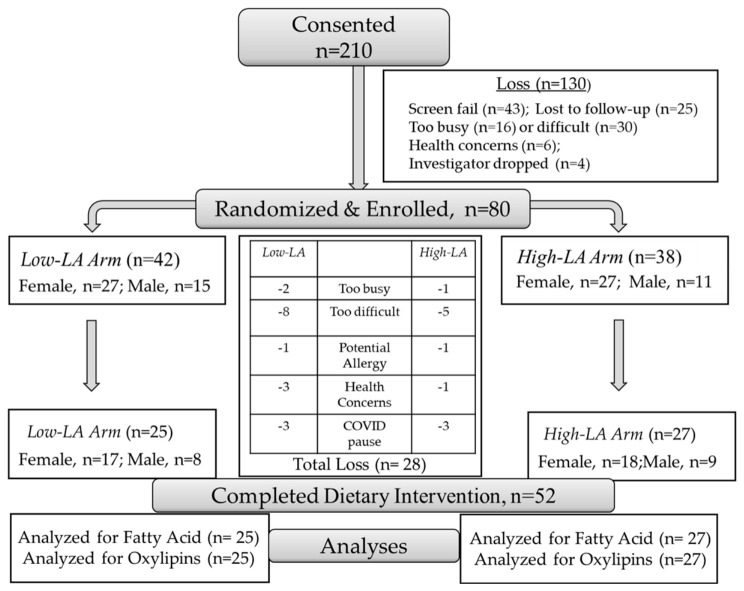
Recruitment and Retention. Healthy, free-living adult participants were recruited for the 12-week dietary intervention. Pre- and post-enrollment numbers reflect the intervention’s challenging nature despite extensive participant education. The counts for gender distribution are reported for those randomized (n = 80) to the intervention and for those completing (n = 52) the entire 12-week intervention.

Figure 2 shows a flow diagram of recruitment and retention status for the study. For all participants, written informed consent was obtained during an initial screening visit. Consented participants who met the inclusion criteria and were deemed candidates for this challenging dietary intervention, based on interviews conducted by the dietary staff using a standard questionnaire addressing the study challenges, were invited to join the intervention phase of the study. Block randomization was used to ensure approximately equal accrual to each sequence within each stratum over time. The Nutrition Coordinator (an RD; L. Easter) performed the randomization using files provided by the study biostatistician and retained the randomization log which was locked in her office. To ensure correct oil usage, the Metabolic Kitchen personnel were blinded and used color-coding for oils and study products to identify and distinguish the two arms. All other study staff and participants were blinded to dietary arm assignment.

### 2.2. Dietary Intervention

The study was designed to accurately capture PUFA dietary exposure [32] and to provide approximately 95% of daily caloric intake from fat. Prior to the dietary intervention, participants underwent a fasting resting metabolic rate (RMR) test using a MGC Ultima CCM™ indirect calorimeter [33]. RDs also collected information on typical eating habits (3-day food log), exercise habits, and food sensitivities. These data were used to estimate the daily meal plan needed to meet the study nutrient goals and the caloric target required to maintain body weight measured at that study visit. A change in more than ±5 lbs in current weight during the intervention phase (based on weekly weights) required adjusting the daily meal plan’s caloric level (typically in 200-calorie increments). Of the 80 participants who began the dietary intervention, the daily caloric goal needed to be adjusted for 20 participants, nearly equally distributed between the two study arms. Gathering information on eating habits and food sensitivities was imperative, as it helped identify participants who would not be suited for participation from a dietary perspective. The study could not accommodate individuals with known allergies to study oils or gluten, those on a vegan diet, those who were lactose intolerant (unless already acclimated to lactase enzyme supplements), or others whose lifestyle or preferences would prevent compliance with the study guidelines. Such participants were eliminated as candidates. Additionally, a formal common-diet run-in period was not included in the study design, which represents a limitation.

#### 2.2.1. Food Grade Oils

Oils are among the largest sources of dietary fat. Because the intervention aimed to manipulate LA exposure in the two study arms, we used a pair of commonly available food-grade oils that differ in LA content. A methodological consideration inherent to dietary fatty acid interventions is that altering the proportion of one fatty acid necessarily displaces another within the total fat energy budget. In the present study, reduced LA exposure was achieved by increasing oleic acid intake. Although this means the two dietary arms represent distinct, broader fatty acid environments rather than differing only in LA, this design was intentional and scientifically justified. Oleic acid is a monounsaturated fatty acid that does not serve as a substrate for the Δ6-desaturase enzyme and does not compete within the PUFA elongation and desaturation pathway that converts LA and ALA to their long-chain derivatives. As such, the reciprocal increase in oleic acid is not expected to directly influence the PUFA metabolic outcomes under investigation.

Naturally occurring safflower variants with seed oils containing low (~13%) and high (~73%) LA were used to manipulate dietary LA exposure (Supplementary Table S1). In the low-LA oil (high-oleic-acid safflower), the oleic acid content was higher, and the LA content was lower. Flaxseed oil, a source of ALA (~56%), was provided in equal amounts to both study arms.

Bulk oils were obtained from commercial suppliers after collecting samples for analysis to evaluate their suitability (see below). A thorough due diligence process was carried out to identify high-quality, food-grade oil products from trustworthy sources [34]. The oil sources were: flaxseed oil (Shape Foods, Inc.; Brandon, MB, Canada); high-oleic safflower oil (Oilseeds International Ltd.; San Francisco, CA, USA, and Arista Industries Inc.; Wilton, CT, USA); and high-linoleic safflower oil (Oilseeds International Ltd.; San Francisco, CA, USA, and Welch, Holme & Clark Co.; Newark, NJ, USA).

#### 2.2.2. Study-Provided Foods and Participant Educational Tools

During the dietary intervention, participants received a wide variety of study foods weekly, prepared with the study oils and providing the majority (>95%) of their daily dietary fat intake (25–30% of calories). All study-supplied foods were prepared, pre-weighed, and packaged by the WFUHS Clinical Research Unit (CRU) Metabolic Kitchen staff under research conditions. Daily smoothies (3–4 flavor recipes offered) served as a vehicle for flaxseed oil, which was common to both arms. This delivery mechanism avoided heat-induced oxidation of the oil. Other provided study foods included study oil-based condiments (salad dressings, mayonnaise, and sauces) and a variety of prepared snack foods (cookies, brownies, granola, sweet/salty snack mixes, hummus), each made with the arm-specific type of safflower oil. Due to the neutral flavor and aroma of the safflower oils, the study products prepared for both arms appeared identical in taste, texture, and smell. A can of PAM™ aerosolized vegetable oil was dispensed, as needed, for participants to use in cooking. The recipes for all provided food items were designed to contribute a standard number of calories to the daily diet.

Participants ordered their preferred study foods weekly. All other allowed foods, including low- or non-fat animal products (with controlled amounts of specific seafoods), grains, fruits, vegetables, fat-free sweets, and non-caloric beverages, were provided by the participants. An individualized meal plan was designed for each participant with input from both the RD and the participant. The participants’ input was essential to best accommodate their typical eating patterns and dietary preferences within the study’s nutrient requirements. The meal plans provided participants with guidance for planning all meals, which in turn facilitated their adherence to this challenging intervention protocol. It was critical for participants to avoid additional fat calories from other food types (dairy, meats, baked goods, etc.) to remain compliant with the study protocol. Regular education and review of food logs were conducted to reinforce appropriate choices and adherence to this criterion. No run-in period on a common diet was employed for this study. Instead, the study RD and her team provided extensive, arm-independent, one-on-one dietary education and counseling as an essential element of the study protocol. RDs provided a variety of assistance, including individualized meal planning, grocery-shopping guides, instructions on interpreting food labels for fat content, guidance on home meal preparation, guidance on dining out, and portion-size estimates. Intensive RD-delivered counseling was provided prior to the start of the dietary intervention and continued weekly during food pick-up visits. Other education, tools, and counseling interactions provided to participants by the RDs included: (a) lists of allowed and disallowed foods, (b) food label reading practice (to select allowed foods and avoid extra fat exposure from grocery store products), (c) use of a food scale (study-provided) to measure portions, (d) sample menu plans, (e) recipes and suggestions to enhance food item acceptability, (f) lists of appropriate menu selections at various types of restaurants, (g) safe food handling procedures, and (h) guided modification of the individualized meal plan, as needed. All dietary information was delivered by the RDs in a uniform manner to all participants.

### 2.3. Biospecimen, Collection, Handling, and Storage

At the initial screening/consenting visit, blood was collected to obtain serum for screening against exclusion criteria (lipid panel, glucose, hs-CRP). The remaining serum was aliquoted and stored at −70 °C for future use. At subsequent visits, a dedicated tube of heparinized blood was collected at each fasting visit during the dietary intervention (weeks 0, 4, 8, and 12). Within 30 min of collection, whole blood was subjected to a zymosan-stimulation assay to evaluate oxylipin generation [35,36,37]. Briefly, whole blood was incubated at 37 °C for 30 min in the presence of phosphate-buffered saline or zymosan (Sigma-Aldrich, St. Louis, MO, USA) suspended in the same buffer (2.5 mg/mL, final concentration). At the end of the incubation period, plasma was collected, aliquoted, blanketed with argon, and stored at −70 °C for oxylipin mass spectrometry analysis (below).

Laboratory staff were blinded to the study arm. All samples for a participant were analyzed in the same batch. If it was necessary to reanalyze a sample, a new aliquot was used to minimize any effects of freeze–thaw cycles.

### 2.4. Measurements

During the dietary intervention, vital signs (blood pressure and resting heart rate) and morphometric measurements (waist and hip circumference, height, body mass index (BMI), and percent body fat) were obtained at 4-week intervals. Participants were weighed weekly by the RD staff during food pickup visits. The primary outcomes were circulating levels of *n*-6 and *n*-3 PUFAs and HUFAs, and secondary outcomes included ratios of circulating *n*-6 and *n*-3 HUFAs, and levels and ratios of ARA- and EPA-derived lipoxygenase-derived oxylipins.

#### 2.4.1. Compliance

Compliance with the intervention was monitored in two ways. Dietary exposure to LA was obtained from weigh-backs of returned, uneaten study foods and from food logs completed by participants in real time and returned to the RD at weekly visits. The food logs captured intake of study-provided foods and self-selected foods and were analyzed using the Nutrition Data System for Research (NDSR), developed at the University of Minnesota Nutrition Coordinating Center [38,39]. During visits, the RD queried participants about any incomplete entries to obtain sufficient detail to calculate nutrient intake. The RD also discussed any significant deviations from the prescribed meal plan, especially those related to fat intake, using these logs, and redirected participants with suggestions for appropriate alternate choices. Cumulative nutrient intake data used to evaluate compliance included the percentages of calories from LA, ALA, total fat, carbohydrates, and protein. The second measure of compliance was circulating fatty acid profiles measured from fasting blood samples obtained at 4-week intervals during the intervention.

#### 2.4.2. Fatty Acid Analysis in Plasma, Oils, and Study Foods

Total plasma fatty acids were analyzed from fasting blood samples. Fatty acids, including LA, ALA, oleic acid (OA), gamma-linolenic acid (GLA), dihomo-gamma-linolenic acid (DGLA), ARA, stearidonic acid (SDA), eicosatetraenoic acid (ETA), EPA, docosapentaenoic acid (DPA), and docosahexaenoic acid (DHA), were analyzed as fatty acid methyl esters (FAME). FAMEs were prepared [40] after alkaline hydrolysis of complex lipids in duplicate samples (100 μL of plasma) in the presence of an internal standard (triheptadecanoin: NuChek Prep, Elysian, MN, USA; included for fatty acid quantification), as previously described [41,42]. Duplicate plasma volumes (100 μL) per participant were assayed, and participant sample sets were processed together in the same batch. Participant sets were processed in a randomized manner.

A standard panel of 25 fatty acids (accounting for 99% of fatty acids in the samples) was quantified by gas chromatography with flame ionization detection (GC-FID) on an Agilent 7890 (Santa Clara, CA, USA) instrument with a DB-23 column (30 m, 0.25 mm ID, 0.25 µm film) fitted with an inert pre-column (1 m, 0.53 mm ID) for cool on-column injection. Briefly, the initial oven temperature (90 °C) was held constant for 3 min; then increased at 10 °C/min to 170 °C and held for 5 min; then increased at 5 °C/min to 220 °C; and then increased at 10 °C/min to 230 °C, held for 3 min before cooling back to 90 °C. The intra-assay coefficient of variation (CV) for FAME analyses was 5.6%, and the inter-assay CV was 12.3%. Data capture and analyses were performed using ChromPerfect (Denville, NJ, USA) software (v6.0.12). The instrument response factor was calculated using external standard sets for quality assurance, and a mixture of known FAMEs was run with each sample set to monitor instrument performance [43]. Individual fatty acids in plasma were expressed as concentrations.

The fatty acid composition of the oil supplements (Supplementary Table S1) was determined by analyzing aliquots of oil diluted in hexane and processed as described above in the presence of the internal standard. For product authentication, individual fatty acids in oil products were expressed as area% (Supplementary Table S1) to facilitate comparison with vendor documentation of the product fatty acid profile.

The integrity of the study oils was evaluated upon receipt, before transferring to the Metabolic Kitchen, and at 5- to 6-month intervals, with additional checks upon return of empty containers from the Metabolic Kitchen. The quality control (QC) testing included routine evaluation of fatty acid profile and oxidation status to ensure that a high-quality product was provided to the Metabolic Kitchen for study food preparation. Additional details regarding the study oil QC testing can be found in Supplementary Information (Figures S1–S3).

The oxidation status of oils was assessed using standard food-industry assays. These included: (a) peroxide value (PV; primary oxidants; iodometric titration assay [44]; European Pharmacopeia 2.5.5); (b) anisidine value (AV; secondary oxidants; colorimetric assay [45]; European Pharmacopeia 2.5.36); and Totox (total oxidation = 2 PV + AV). Oil was considered out of spec when the Totox value was ≥30. Such oil containers were removed from use and discarded.

The fatty acid profiles of the study food items were also monitored qualitatively throughout the study as a quality control measure to ensure that the correct oils were used for the intended arm. A finite weight of food product (50–150 mg, depending on the oil content of the food item) was processed as described above for FAME analysis in the absence of the internal standard. It was not possible to perform an oxidation assay on the study food items due to interfering substances in these samples.

#### 2.4.3. Oxylipin Analysis

Oxylipins were extracted from plasma (100 µL) in methanol (LC-MS grade) in the presence of deuterated internal standards. Briefly, methanolic extracts were diluted with 9 volumes of water and centrifuged at room temperature to remove precipitated proteins. The supernatant was subjected to solid-phase extraction (Phenomenex (Torrance, CA, USA); Strata X-33 µm Polymeric Reverse Phase; #8B-S100-EBJ) using a vacuum manifold. The column was preconditioned (1 mL methanol followed by 1 mL water) before loading the sample supernatant. After the sample slowly eluted from the column, the column was washed once with 10% methanol. Oxylipins were eluted with 1 mL methanol, and the eluent was collected in 1.5 mL screw-cap tubes, blanketed with argon, and stored at −80 °C. Samples were prepared for analysis by drying under a stream of nitrogen and redissolving in 50 µL water:methanol (1:1). Sample extraction and analysis began near the end of the study, and both sample preparation (by participant sample set) and analysis order were randomized to minimize batch effects.

Separation of the analytes was performed on a 150 × 3 mm × 2.6 um Phenomenex Kinetex C8 column (PN: 00F-4497-Y0). The liquid chromatography system consisted of Shimadzu (Kyoto, Japan) LC-40D x3 solvent delivery modules, a DGU-405 degasser, an SCL-40 system controller, and a CTO-40C column oven. Analytes were eluted with a mobile phase consisting of Fisher OmniSolv water with 0.1% formic acid (Fisher Scientific; Waltham, MA, USA) as solvent A and 100% Fisher OmniSolv acetonitrile as solvent B. Solvent flow was set to 0.4 mL/min, starting at 30% B. The gradient was increased to 95% B over 9 min and then held until 11 min. The concentration was then decreased back to 30% B at 11.1 min and held until the method ended. The mass spectrometer, an AB SCIEX 7500 triple quadrupole (SciEx, Framingham, MA, USA), was run in negative ion mode using nitrogen gas for ion generation under the following conditions: Ion source gas 1 and 2 were set at 60 and 90 psi, respectively; the curtain gas was set at 40 psi; and the CAD gas was set to 12. The source temperature was set to 350 °C. Ion spray voltage was set to 2000 V. A list of authentic oxylipin compounds used for quantification, their mass transitions, and the deuterated internal standards are shown in Supplementary Table S2. The intra-assay coefficient of variation (CV) for oxylipin analyses was 8.9%, and the inter-assay CV was 11.7%. The zymosan-stimulated oxylipin generation was calculated as the abundance (pg/mL) in the plus zymosan condition minus that in the buffer control condition and was not intended to assess circulating oxylipin levels. The resultant zymosan-stimulated oxylipin data were cleaned of zero or negative values. Oxylipins with <15% missing data (and therefore 85% usable data) were used for further analyses and considered informative. This data cleaning did not differ by arm.

#### 2.4.4. Clinical Analysis

A dedicated tube of blood for serum preparation was collected at each fasting study visit (the initial screening/consenting visit and each of the four monthly visits during the dietary intervention). Routine cardiometabolic and inflammatory biomarkers were measured as secondary outcomes. These included: (1) glucose (enzymatic assay) measured in serum from fasting blood; (2) high-sensitivity *C*-reactive protein (hsCRP; immunochemoluminometric assay); and (3) serum lipids (total cholesterol, triglycerides, HDL-, VLDL-, and LDL-cholesterol; enzymatic assays). These endpoints were analyzed by a qualified clinical laboratory (Lab Corp, Burlington, NC, USA).

### 2.5. Data Analysis

The primary outcomes of this study were the differences between the High- and Low-LA arms at the 12-week time point for four fatty acids: LA, ARA, EPA and DHA. The comparisons at the other time points for these FAs as well as the remaining FAs served as secondary endpoints. Other secondary endpoints included several ratios: ARA/EPA, ARA/DHA, ARA/DGLA, and the oxylipin ratios 5-HETE/5-HEPE and 5-HETE/5-HETrE.

Statistical analyses of baseline characteristics and dietary compliance were conducted in GraphPad (version 5). Statistical comparisons were made between arms using a 2-tailed *t*-test.

A mixed-model framework was used to analyze the FA and oxylipin values and ratios. Analyses were conducted in R (4.4.0) and RStudio (2023.06.1+524) using the lmer function in the lmerTest package (3.1-3), where we fit the following model for each outcome:

outcome~Age + Sex + studyWk ∗ Arm + baseline_value + (1|ID),

where each participant had a random intercept and REML was used for estimation. The time variable, studyWk, was coded as a factor and models were adjusted for baseline due to substantial differences between arms in some cases. Differences between the two arms at each time point (i.e., weeks 4, 8, and 12) were estimated and tested using the estimated marginal means (least-squares means) approach as implemented in the R package emmeans (1.11.0). The Bonferroni-Holm method was used to adjust *p*-values for the four primary outcomes. For all other secondary outcomes (including other time points for the four primary FAs), we used the Benjamini–Hochberg procedure to control the false discovery rate (FDR). We present full results in Supplementary Tables S4 and S5.

Given the mechanistic nature of the study and its focus on biochemical outcomes contingent on verified dietary exposure, data were analyzed following the intention-to-treat (ITT) approach with all randomized participants included regardless of protocol adherence. Below detection values for low abundance FAs and oxylipins were replaced with a small value (LOD/2) in a small number of cases. Otherwise, participants contributed complete primary and secondary endpoint data at all time points at which they were measured and no data imputation was necessary or performed.

## 3. Results

### 3.1. Characteristics of the Study Population

The target study population consisted of healthy adults. As shown in Figure 2, 80 consenting participants met eligibility criteria and were enrolled in the dietary intervention phase. After randomization, 16 participants withdrew, primarily because of scheduling constraints or difficulty adhering to the intervention protocol. Notably, participants who reported difficulty maintaining mindful daily dietary tracking were more likely to show poor compliance. Additional attrition resulted from suspected study-related food allergy (n = 2), emerging health concerns unrelated to the intervention (n = 4), and an institutional pause in research activities during the COVID-19 pandemic (n = 6). Ultimately, 52 participants completed the full 12-week dietary intervention: 25 in the Low-LA arm and 27 in the High-LA arm.

Baseline demographic and clinical characteristics of the 80 randomized participants are shown in Table 1. The two intervention arms were comparable in initial sample size, sex distribution, and racial composition. Among age, anthropometric measures, cardiovascular indices, and standard clinical laboratory parameters, only fasting HDL cholesterol and fasting glucose differed modestly between groups at baseline.

Importantly, baseline (Day 0) fasting plasma *n*-6 and *n*-3 PUFA and HUFA concentrations (mg/dL) were similarly distributed across the two study arms (Supplementary Table S3). Furthermore, plasma PUFA-to-HUFA ratios, used as surrogate markers of enzymatic activity in the shared elongation and desaturation biosynthetic pathway, did not differ between groups at baseline, supporting metabolic equivalence before dietary intervention.

### 3.2. Impact of Dietary Intervention

Manipulating dietary LA exposure to determine PUFA-to-HUFA metabolism in healthy, free-living adults posed a substantial design challenge, as typical U.S. dietary intake of LA ranges from approximately 6–9% of total energy. Accordingly, a dietary intervention was designed to sustain markedly divergent LA exposures, targeting 10% of total energy in the High LA arm and 2.5% in the Low LA arm.

**Figure 3:**
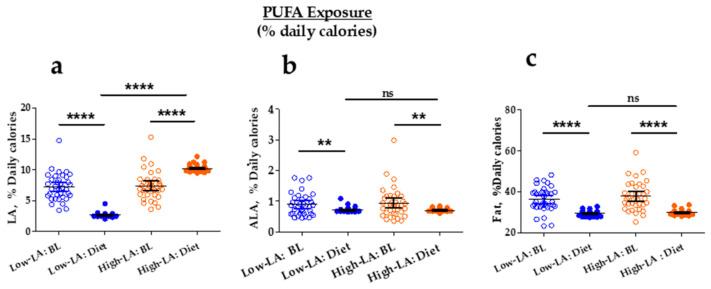
Effects of Dietary Intervention on LA Exposure. Dietary intakes of LA (**a**), ALA (**b**), and total fat (**c**) are expressed as a percentage of daily caloric intake. Pre-intervention baseline (BL) estimates were derived from three-day food logs collected before the intervention (open symbols). Compliance during the intervention was assessed using weekly diet logs (closed symbols). Data represent means ± 95% CI; group differences were assessed by two-tailed Student’s *t*-test. Significance levels: ns, not significant; **, *p* < 0.01; ****, *p* < 0.0001.

The two dietary exposure targets were achieved and maintained throughout the 12-week intervention period (Figure 3), indicating that the education and support provided by the RDs were effective in ensuring compliance and preventing the use of “hidden fats”. LA intake during the intervention was 2.65 ± 0.06% energy in the Low-LA arm and 10.21 ± 0.09% energy in the High-LA arm (Figure 3a). Thus, 97% of participants in the Low-LA arm were compliant with the arm’s target. For the High-LA arm, 78% were compliant with the LA target (10% calories as LA), with the remainder slightly lower (n = 1, 9%) or higher (n = 7, 11–12%). Pre-intervention (baseline) LA intake did not differ between study arms (Figure 3a, open symbols). Following initiation of the intervention (Figure 3a, closed symbols), LA exposure was significantly reduced in the Low-LA group and significantly increased in the High-LA group, confirming successful execution of the intended dietary manipulation.

In contrast, ALA exposure (<2.5% EN) remained constant across both dietary arms (Figure 3b; closed symbols). Notably, total fat-calorie intake did not differ between the two dietary arms during the intervention. However, both arms showed an approximately 20% decrease in fat-calorie consumption during the intervention compared with baseline (Figure 3c; closed symbols). By the end of the intervention, there were no arm-dependent differences in body weight, body fat, or BMI, as total daily calories were not different between arms. These findings likely reflected broader dietary behavioral changes associated with study participation and dietary counseling. This phenomenon is commonly observed in controlled feeding and dietary guidance trials. Importantly, this reduction was symmetric across arms and did not differ significantly between the high-LA and low-LA groups at any intervention time point.

**Figure 4:**
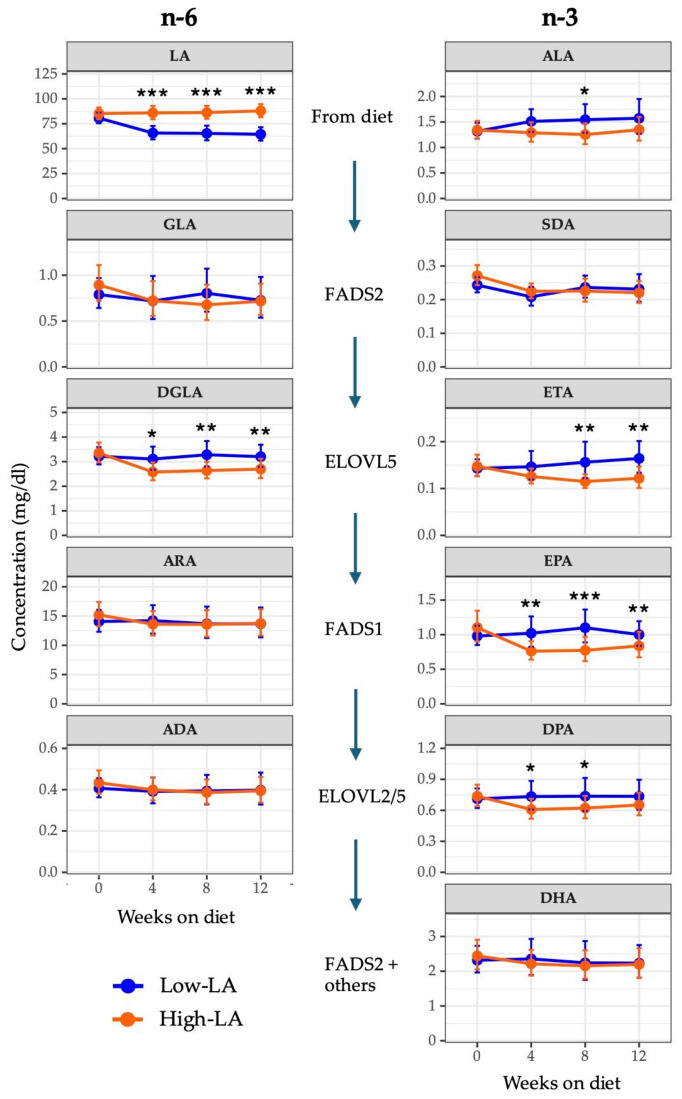
Plasma PUFA and HUFA Concentrations Across Their Biosynthetic Metabolic Pathway. Fasting concentrations (mg/dL) for paired *n*-6 and *n*-3 PUFA-HUFA at each enzymatic step of the pathway are shown. Adrenic acid (ADA). Data are presented as geometric means ± 95% CIs. The two arms were compared at each time point using mixed-effects models adjusted for age, sex, study week (factor), arm, their interaction, and baseline value. *p*-values are represented as *, *p* < 0.05; **, *p* < 0.01; ***, *p* < 1 × 10^−3^.

Dietary compliance was further validated using fasting plasma PUFA concentrations (mg/dL). As shown in Figure 4, elevated dietary LA exposure in the High-LA arm led to significantly higher circulating plasma LA concentrations than in the Low-LA arm at weeks 4, 8, and 12 (Supplementary Table S4). In contrast, controlled ALA intake produced no between-arm differences in fasting plasma ALA concentrations throughout the intervention. Importantly, the circulating LA and ALA levels closely mirrored dietary exposure derived from food record compliance data.

### 3.3. Impact of LA Exposure on PUFA to HUFA Biosynthesis

As outlined in the Introduction, the PUFA-to-HUFA biosynthetic pathway proceeds through alternating desaturation and elongation steps, catalyzed sequentially by FADS2 (Δ6-desaturase), ELOVL5 (elongase 5), and FADS1 (Δ5-desaturase). Because *n*-6 and *n*-3 PUFAs share the same enzymatic machinery, this pathway is a critical node in the metabolic competition between the two fatty acid families. A significant arm-dependent divergence was observed at both the ELOVL5 and FADS1 steps of the *n*-3 branch, resulting in markedly lower plasma concentrations of the *n*-3 HUFAs, ETA and EPA in the High-LA arm (Figure 4, Supplementary Table S4). This suppression of *n*-3 HUFA production is consistent with elevated LA competitively displacing *n*-3 substrates at key enzymatic steps. Plasma concentrations of DPA were also significantly lower at weeks 4 and 8 (Figure 4, Supplementary Table S4) in the High-LA group, suggesting that the suppressive effect of elevated LA may extend further along the *n*-3 elongation pathway.

In contrast, the *n*-6 HUFA ARA did not differ between arms (Figure 4), indicating that approximately 2.5% of energy from LA is sufficient to sustain near-maximal flux through the *n*-6 branch of the pathway. There was a small but significant increase in DGLA at 10% energy from LA (Figure 4, Supplementary Table S4). The absence of further increases in ARA at 10% energy suggests saturation of *n*-6 HUFA biosynthesis at the lower intake level.

**Figure 5:**
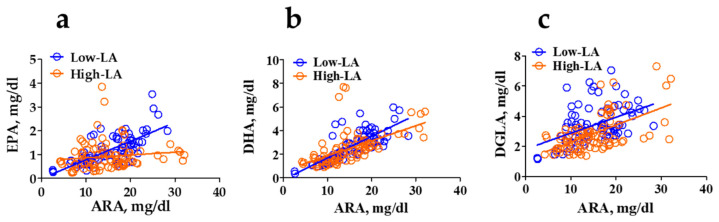
Plasma EPA Concentrations are Arm-Dependent. Regression analyses of plasma EPA with ARA (**a**), DHA with ARA (**b**), or DGLA with ARA (**c**) concentrations (mg/dL) for intervention weeks (4, 8, 4, 8, 12) are shown by study arm. The slopes of the regression lines for EPA were significantly different: EPA-ARA (F = 27.85, *p* < 0.0001), but much less so for DHA-ARA (F = 4.48, *p* = 0.036) and not significant for DGLA (F = 0.084, *p* = 0.773).

Regression analyses of EPA (Figure 5a), DHA (Figure 5b), and DGLA concentrations (Figure 5c) as a function of ARA concentrations across study weeks 4–12 revealed a markedly LA-dependent relationship for EPA (F = 27.85; *p* < 0.0001), but not for DHA (F = 4.48; *p* = 0.036) or DGLA (F = 0.084, *p* = 0.773). This analysis further supported the conclusion that elevated dietary LA potently suppresses EPA accumulation relative to ARA.

**Figure 6:**
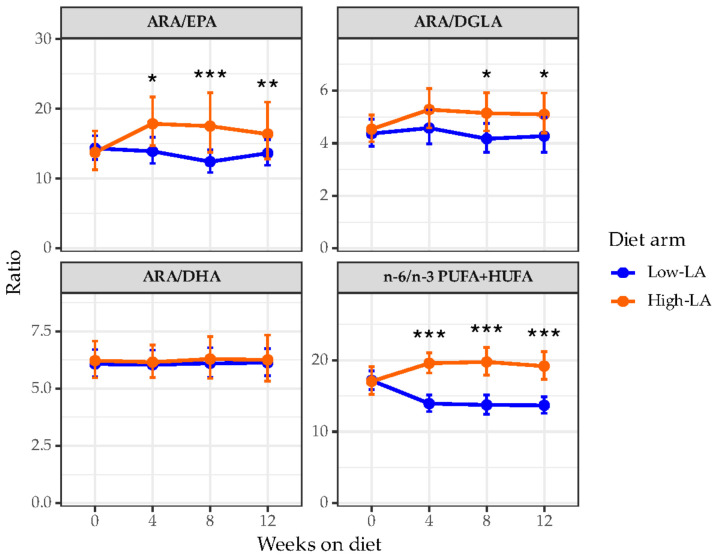
Impact of Dietary LA on HUFA Ratios. HUFA ratios of ARA to EPA, ARA to DHA, ARA to DGLA, or total *n*-6/*n*-3 were calculated from fatty acid concentrations (mg/dL). Data are shown as means. Statistical comparisons were made using mixed-effects models adjusted for age, sex, study week (factor), arm, their interaction, and baseline value. *p*-values are represented as follows: *, *p* < 0.05; **, *p* < 0.01; ***, *p* < 1 × 10^−3^.

Consistent with the marked reduction in EPA relative to ARA, the plasma ARA/EPA ratio was significantly elevated in the High-LA arm (Figure 6, Supplementary Table S5). There was also a small but significant difference in the ARA/DGLA ratios between arms at 8 and 12 weeks (Figure 6). Importantly, the arm-driven shift in *n*-3 HUFA synthesis was consistent over time, as shown by the total *n*-6 to *n*-3 PUFA + HUFA ratio (Figure 6, Supplementary Table S5). This ratio was markedly higher in the High-LA arm, reflecting both the elevated *n*-6 substrate load and the suppression of *n*-3 HUFA production along the shared biosynthetic pathway. This broad shift in the *n*-6 to *n*-3 balance highlighted that the metabolic consequences of high dietary LA extend beyond any single fatty acid.

### 3.4. Impact of Dietary LA in Oxylipin Generation

Most assessments of oxylipins in human disease have focused on circulating species measured in plasma or serum, where oxylipins exist in both non-esterified (free) and esterified (phospholipid- and lipoprotein-bound) pools [46,47,48]. However, given the large number of enzymatic (LOX, COX, CYP) and non-enzymatic pathways that generate oxylipins, their rapid turnover and metabolism, and the dynamic interconversion between esterified and free pools, it remains uncertain how well static circulating concentrations reflect pathway flux or functional inflammatory potential.

**Figure 7:**
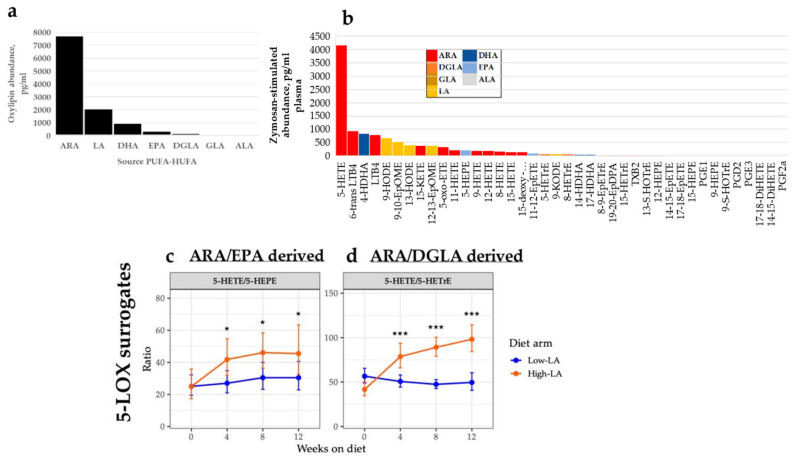
Abundance of Oxylipins Generated by Zymosan-Stimulation of Whole Blood. The abundance of oxylipins (pg/mL) generated from zymosan-stimulated whole blood was ranked by PUFA or HUFA source (**a**). The individual oxylipins (pg/mL plasma; mean values) generated are shown (**b**; 15-deoxy-PGJ2 truncated to ‘15-deoxy…’). The oxylipin ratios of ARA-derived 5-HETE to EPA-derived 5-HEPE and ARA-derived 5-HETE to DGLA-derived 5-HETrE are shown in (**c**) and (**d**), respectively. Significant differences by *p*-value are represented as follows: *, *p* < 0.05; ***, *p* < 1 × 10^−3^.

To address this limitation, the current study evaluated the impact of dietary LA exposure on oxylipin production capacity using a zymosan-stimulated whole-blood assay, thereby capturing integrated leukocyte-driven enzymatic responses under a standardized inflammatory challenge, with a particular emphasis on 5-lipoxygenase (LOX)-derived products. Targeted LC–MS/MS profiling yielded high-quality quantitative data for 40 oxylipin species. Among oxylipins generated in the zymosan-stimulated assay, 35% (14 species) were derived from ARA, 25% (10 species) from EPA, 15% (6 species) from LA, 10% each from DGLA and DHA, and one species each from ALA and GLA (Figure 7a).

Quantitative analyses of individual species confirmed the predominance of a 5-lipoxygenase (5-LOX)-mediated response, with marked enrichment of 5-hydroxyeicosatetraenoic acid (5-HETE) among ARA-derived products (Figure 7b). Among EPA-derived metabolites, 5-hydroxy-eicosapentaenoic acid (5-HEPE) was the most abundant species. Low-abundance oxylipins (<50 pg/mL) are presented in Supplementary Figure S4. 5-LOX also utilizes DGLA, and its product is 5-hydroxy-6E,8Z,11Z-eicosatrienoic acid (HETrE). The 5-HETE/5-HEPE ratio (ARA-derived/EPA-derived) and 5-HETE/5-HETrE ratio (ARA-derived/DGLA-derived) reflect the utilization of *n*-3 versus *n*-6 HUFA oxylipin precursors. Both ratios were consistently higher in the High LA arm (Figure 7c,d), indicating significantly elevated 5-LOX production from ARA compared to both EPA and DGLA.

## 4. Discussion

This controlled dietary intervention demonstrates that LA, within the ranges commonly consumed in modern diets, is a dominant regulator of the human PUFA-to-HUFA metabolic network. Increasing LA from ~2.5% to ~10% of total energy markedly suppressed endogenous *n*-3 HUFA synthesis, reduced circulating EPA concentrations, increased the ARA/EPA ratio, and shifted leukocyte 5-LOX metabolism toward preferential formation of ARA-derived 5-HETE during an inflammatory challenge. These findings provide direct human evidence that LA intake not only contributes to membrane composition but also exerts competitive control over a shared enzymatic pathway, with downstream consequences for inflammatory signaling capacity.

### 4.1. Major Challenge of This Study

The *n*-6 and *n*-3 PUFA families share a finite set of desaturation and elongation enzymes. Although substrate competition within this pathway has been inferred from in vitro and animal studies [26,27], human data across physiologically relevant LA exposures have remained sparse. A major challenge in conducting such studies is the dietary control required to achieve meaningfully distinct LA exposure levels, particularly at the low end of the range. The 2.5% energy LA target achieved in the Low-LA arm of this study represents a substantially reduced LA intake compared to typical Western diets and required considerable dietary intervention to sustain.

The logistical demands of achieving this level of dietary control were considerable. Rigorous compliance monitoring and intensive participant support were necessary throughout the intervention period to maintain adherence to the prescribed diets, and the study required substantial staffing resources to achieve this. Despite these efforts, 16% of participants withdrew from the study, predominantly from the Low-LA arm, reflecting the challenge of making lifestyle changes to adhere to substantially modified dietary patterns during the intervention period. The need to maintain adequate staff-to-participant ratios to ensure dietary compliance necessarily constrained the study’s overall sample size to n = 80.

### 4.2. LA as a Competitive Metabolic Gatekeeper

Here, ~2.5% energy from LA was sufficient to sustain circulating ARA levels, suggesting that near-maximal *n*-6 HUFA synthesis occurs at relatively modest LA intake. In contrast, elevating LA to ~10% energy did not increase ARA, suggesting that desaturation may be operating at or near Vmax under baseline Western Diet conditions and that further increases in dietary LA cannot drive additional flux through the pathway. At ~10% energy LA, the *n*-3 branch was suppressed, with significant reductions in total plasma concentrations of ETA, EPA, and DPA, while DHA remained unchanged. This aligns with earlier studies that challenged humans with LA exposure, evaluated EPA in plasma lipid fractions [49,50], and examined the established enzymatic loci of *n*-6 and *n*-3 PUFA to HUFA competition [51].

This asymmetric response reveals a hierarchical architecture: additional LA does not further increase ARA production but instead redirects flux away from *n*-3 HUFA synthesis. Within the intake range studied, LA therefore functions less as a simple nutrient and more as a competitive gatekeeper of pathway allocation. Importantly, these data suggest that LA intake within the MWD may exceed the threshold required for *n*-6 sufficiency and, perhaps more importantly, constrain endogenous *n*-3 HUFA production.

### 4.3. Functional Recalibration of Inflammatory Signaling

Shifts in HUFA levels were paralleled by changes in oxylipin-generating capacity. Using a zymosan-stimulated whole-blood assay, we captured integrated leukocyte enzymatic responses under standardized activation conditions. The dominance of 5-LOX–derived products reflects the central role of this pathway in acute inflammatory signaling. Because 5-LOX uses ARA, EPA, and DGLA as substrates, product ratios directly index enzymatic substrate competition. Elevated LA intake increased both the 5-HETE/5-HEPE (ARA-derived/EPA-derived) and 5-HETE/5-HETrE (ARA-derived/DGLA-derived) ratios, indicating preferential metabolism of ARA under high LA exposure. The impact of the high-LA diet on the 5-HETE/5-HETrE ratio was unexpected and may reflect compartmentalization of 5-LOX substrates in response to high LA levels.

### 4.4. Rethinking “Total PUFA”

ALA intake and total PUFA exposure were held constant across arms, yet marked divergence emerged in HUFA composition and oxylipin output. These results challenge the prevailing practice of grouping PUFAs as a single functional class [52]. These data reveal that total PUFA quantity does not predict metabolic outcome; rather, subclass competition determines pathway flux. Within the examined intake range, increasing LA did not increase ARA abundance but disproportionately suppressed *n*-3 HUFA generation, thereby altering the *n*-6/*n*-3 ratio. This competitive topology implies that the metabolic effects of LA cannot be inferred solely from cholesterol reduction or total PUFA metrics. Network redistribution effects must be considered.

### 4.5. EPA as the Metabolically Sensitive Node

The selective suppression of EPA, while DHA showed a non-significant trend toward reduction, identifies EPA as the most diet-responsive node in the endogenous *n*-3 pathway under competitive LA exposure. DHA synthesis and concentrations require additional elongation, desaturation, and peroxisomal processing steps [53]. DHA concentrations may also be more strongly influenced by DHA exposure, which may buffer against substrate competition. Alternatively, DHA pools may be preferentially conserved due to structural demands. The dissociation between EPA and DHA responses suggests that endogenous EPA may be particularly vulnerable to high LA exposure, with potential implications for interpreting circulating EPA as a biomarker of dietary fatty acid balance and inflammatory capacity.

### 4.6. Context Within the Ongoing LA Debate

The role of dietary LA in cardiometabolic health remains debated. Observational and interventional studies have frequently reported inverse associations between LA intake and LDL cholesterol or cardiovascular risk, leading to recommendations favoring replacing saturated fat with *n*-6 PUFAs [13,14,15,16,17]. However, such analyses rarely account for competitive interactions within the shared PUFA-to-HUFA pathway or for downstream redistribution of *n*-3 substrate flux. The present findings do not directly address clinical outcomes; rather, they identify a mechanistic effect of LA on metabolic network allocation. Within physiologically relevant intake ranges, increasing LA does not further increase ARA abundance but instead selectively constrains endogenous *n*-3 HUFA synthesis and shifts oxylipin generation toward ARA-derived products. These results suggest that the metabolic consequences of LA exposure cannot be fully inferred from selected clinical metrics alone and underscore the importance of considering the topology of competitive pathways when interpreting dietary PUFA data.

These mechanistic considerations are particularly relevant to socioeconomically disadvantaged populations. Low-income communities in the United States rely disproportionately on ultra-processed foods, which are predominantly formulated with high-LA seed oils because of cost, availability, and constraints in the food environment. Food insecurity and SNAP participation are both associated with higher consumption of ultra-processed foods, with adults experiencing very low food security consuming the highest proportions [10,11,12]. Simultaneously, participants with lower educational attainment and income had significantly lower *n*-3 PUFA and HUFA intakes and fish intakes across NHANES 2003–2014 survey cycles [54]. This consistent pattern suggests that socioeconomically disadvantaged populations are at heightened risk for lower *n*-3 HUFA intake, which may further contribute to health disparities. This convergence of high LA exposure and low *n*-3 PUFA and HUFA intake is the dietary environment in which the competitive pathway suppression of EPA synthesis demonstrated here would be expected to be most consequential.

### 4.7. Strengths and Limitations

This study integrates controlled dietary manipulation, objective validation of biochemical compliance, quantitative lipidomics, and functional oxylipin profiling under standardized immune activation. Isolating LA as the primary manipulated variable strengthens causal inference. Limitations include a moderate sample size, a 12-week duration, reliance on circulating lipids, and in vitro rather than in vivo assessments. Whether sustained LA-driven flux redistribution influences long-term disease trajectories remains to be determined. Additionally, the FADS1/2 genotype likely modulates the magnitude of individual responses to the dietary LA manipulation. The present study was not powered to stratify by FADS genotype. We feel this should be a priority for future investigations.

## 5. Conclusions

These data demonstrate that within physiologically relevant intake ranges, dietary LA exerts competitive control over the shared PUFA desaturation and elongation pathway, selectively suppressing endogenous *n*-3 HUFA synthesis, including EPA, ETA, and DPA, while biasing leukocyte 5-lipoxygenase metabolism toward ARA-derived lipid mediators. Collectively, these findings establish that dietary fatty acid composition is a determinant of metabolic flux through a common enzymatic network. Importantly, these alterations have the capacity to systematically recalibrate the balance of pro- and anti-inflammatory eicosanoid signaling through substrate-level competition without requiring changes in total fat intake or direct supplementation with long-chain *n*-3 PUFAs.

## Figures and Tables

**Table 1:** Baseline Demographics. Baseline demographics of the study cohort (n = 80) at week 0 of the dietary intervention are shown by dietary intervention arm. Data are presented as mean (with 95% confidence intervals). Data were analyzed using a 2-tailed *t*-test, and significant arm differences at baseline are indicated by a bolded *p*-value.

Study Arm	Low-LA	High-LA	*p*-Value
**n**	42	38	
**female (%)**	63.3	71.1	
**EAm (%)**	88.1	92.1	
**Age**	38.9 (35.3, 42.5)	36.8 (32.9, 40.6)	0.3426
**height, cm**	170.8 (168.3, 173.4)	170.2 (167.2, 173.1)	0.3094
**weight, kg**	72.4 (68.9, 75.9)	69.2 (66.0, 72.4)	0.1197
**Body fat, %**	28.9 (26.0, 31.9)	26.8 (23.9, 29.6)	0.2658
**BMI**	24.7 (23.8, 25.6)	23.7 (24.0, 24.5)	0.0863
**Waist, cm**	77.2 (74.2, 80.2)	73.4 (70.9, 75.9)	0.0684
**Hip, cm**	98.8 (96.6, 101.0)	95.8 (95.8, 97.7)	0.1058
**Waist/Hip ratio**	0.781 (0.76, 0.80)	0.766 (0.75, 0.78)	0.4437
**BP, systolic**	114.5 (110.9, 118.1)	113.2 (108.6, 117.9)	0.6680
**BP, diastolic**	69.5 (66.7, 72.4)	68.9 (65.7, 72.2)	0.7392
**Resting heart rate**	64.7 (61.6, 67.7)	67.5 (63.5, 71.5)	0.4845
**Total Cholesterol, mg/dL**	173.3 (163.4, 183.2)	177.2 (165.2, 189.2)	0.7359
**HDL, mg/dL**	56.3 (52.4, 60.2)	65.5 (60.4, 7.06)	**0.0074**
**VLDL, mg/dL**	14.7 (13.0, 16.4)	16.7 (12.9, 260.6)	0.9961
**LDL, mg/dL**	102.3 (93.1, 111.6)	95.2 (85.4, 105.0)	0.3884
**Triglyceride, mg/dL**	73.8 (64.8, 82.9)	82.7 (64.0, 101.5)	0.9693
**Glucose, mg/dL**	92.5 (92.5, 94.8)	87.6 (85.9, 89.4)	**0.0022**
**hsCRP, mg/dL**	1.7 (0.29, 3.16)	0.8 (0.58, 1.03)	0.1539

## Data Availability

The de-identified data presented in this study are available upon request to the corresponding author unless the study has been closed at the level of the Institutional Review Board.

## Supplementary Materials

The following supporting information can be downloaded at: https://www.mdpi.com/article/10.3390/nu18111814/s1, **Table S1: Fatty Acid Profile of Study Oils.** The fatty acid composition of the naturally occurring safflower oils were used to manipulate LA exposure. Flax seed oil served as the source of ALA. The data is presented as area%. The measured fatty acids account for >99% of those in the oils. Bolded fatty acids indicated those targeted by the dietary manipulation. **Table S2: Oxylipin Authentic Standards and Internal Standards.** All authentic oxylipin compounds used to generate standard curves for quantification were obtained from Caymen Chemical (Ann Arbor, MI, USA). Mass transitions used for analyses are shown Deuterated Internal standards were also obtained from Caymen Chemical. Table S3: Baseline Plasma PUFA-HUFA Profile by Arm. Baseline (Week 0 of intervention) plasma PUFA, HUFA and MUFA concentrations (mg/dL) by study arm and ratios are shown. Data are presented as mean values with the 95% confidence interval. Data from the study arms were analyzed by 2-tailed *t*-test with the resultant *p*-value stated. **Figures S1–S3: Fatty Acid Quality Control Testing.** Figure S1: The flax seed oil was obtained from a single supplier in four shipments. None of the flax seed oil batches failed quality control (QC; **Supplementary Figure S1**) testing. Two testing events (June 2020, and June 2021 assesing near empty bottles returned for the Metabolic Kitchen and destined for discard, did show TOTOX values approaching the upper limit (max = 30). The alpha-linoleic acid (ALA) content was consistent across all bottles. Figure S2: The low-LA safflower oil (**Supplementary Figure S2**) was obtained in 5 shipments (15 5-gallon pails), of which two pails failed QC testing at receipt and another pair failed prior to transfer to the Metabolic Kitchen (indicated in black boxed area) and were therefore discarded. It became necessary to change suppliers in 2020 due to difficulties obtaining product from the supplier. The vetting of new suppliers and due diligence of their product were handled with the rigor demanded by the goal of identifying high quality food-grade products. The linoleic acid (LA; middle panel) and oleic acid (OA; right panel) profiles of the oil batches and across suppliers were consistent. Figure S3: The High-LA safflower oil was obtained in 6 shipments (16 5-gallon pails). The oil product was subjected to QC testing (**Supplementary Figure S3**) over time. Those pails failing QC testing are noted in the black-boxed area in the leftmost panel (Oxidation status). Among the oil pails received, 4 pails failed QC testing at receipt, which was attributed to container damage, and were therefore discarded. The prolonged COVID-induced study pause necessitated additional QC testing prior to the study resuming. As a result, a third pail failed this QC testing in 2020 and was discarded. A fourth pail was discovered to have failed QC testing before its planned transfer to the Metabolic Kitchen. It was discarded. The next pail in line was found to be satisfactory and taken to the Metabolic Kitchen for use. It also became necessary to change suppliers in 2019 due to difficulties obtaining product from the initial supplier product discontinuation. The LA (middle panel) and OA (right panel) profiles of the oil batches and across suppliers were consistent. Figure S4: Low Abundance Oxylipins Generated by Zymosan-Stimulated in Whole Blood. This figure shows an expanded portion of the right side of **Figure 7b** to highlight the lower abundance (>50 pg/mL) oxylipins generated during the whole blood stimulation of immune cells by the phagocytic stimulus, zymosan. **Table S4: Mixed-model results for all primary and secondary FA outcomes.** Outcomes were log-transformed and adjusted for baseline, age, sex, study week, treatment arm, and the study week × arm interaction, with a random intercept for participant ID. Time was modeled as a factor due to the non-linear appearance of multiple FA trajectories. *p*-values correspond to the contrast testing for a difference between the two arms at each time point. **Table S5: Mixed-model results for the PUFA-HUFA and Oxylipin ratio outcomes (secondary outcomes).** Mixed-model results for the ratio outcomes (secondary outcomes). Ratios were log-transformed and adjusted for baseline, age, sex, study week, treatment arm, and the study week × arm interaction, with a random intercept for participant ID. Time was modeled as a factor due to the non-linear appearance of multiple FA trajectories. *p*-values correspond to the contrast testing for a difference between the two arms at each time point.
