# Supplementary material for: Effect of Dietary Linoleic Acid Intake on Eicosapentaenoic Acid Status and Lipoxygenase-Mediated Oxylipin Biosynthesis in Healthy Adults: A Randomized Controlled Trial
Source: Nutrients. 2026 Jun 4;18(11):1814. doi: 10.3390/nu18111814 (PMC13259401; doi:10.3390/nu18111814)
Supplement: Supplementary file 1 [file nutrients-18-01814-s001.zip › nutrients-4265303-supplementary.pdf]

Supplemental Information for
Sergeant et. al., Impact of Dietary Linoleic Acid Intake on Eicosapentaenoic Acid Status and Lipoxygenase Oxylipin Metabolism in Healthy Adults: A Randomized Controlled Trial

Supplemental Table S1: Fatty Acid Profile of Dietary Study Oils. The fatty acid composition of the naturally occurring safflower oils were used to manipulate LA exposure. Flax seed oil served as the source of ALA. The data is presented as area%. The measured fatty acids account for >99% of those in the oils. Bolded fatty acids indicated those targeted by the dietary manipulation.

		**Study Oil FA Profile (area%)**		
		Safflower		
**Common Name**	**FA**	**Low-LA**	**High-LA**	**Flaxseed**
Myristolate	C14:0	<0.1	<0.1	<0.1
Palmitic	C16:0	5.4	7.2	5.5
Palmitoleic	C16:1	0	0	<0.1
Stearic	C18:0	2.7	3.5	4.2
**Oleic (OA)**	C18:1 n-9c	**77.4**	**14.5**	**18.8**
Vaccenic	C18:1 n-7c	0.5	0.5	0.5
**Linoleic (LA)**	C18:2w n-6	**12.8**	**73.1**	**14.5**
γ-Linolenic	C18:3 n-6	0.2	0.2	0.1
**α-Linolenic (ALA)**	C18:3 n-3	**0.2**	**0.3**	**56.2**
Stearidonic	C18:4 n-3	<0.1	0	<0.1
Arachidic	C20:0	0.3	0.1	<0.1
Gondoic	C20:1 n-9	0.2	<0.1	<0.1
Behenic	C22:0	0.1	0.1	<0.1
Erucic	C22:1 n-9	0	<0.1	0.0
Nervonic	C24:1 n-9	0.1	0.1	0.0
Others		<0.1	<0.1	<0.1
**Total**		99.9	99.8	99.9

Supplemental Figures S1-S3

Fatty Acid Quality Control Testing

The flax seed oil was obtained from a single supplier in four shipments. None of the flax seed oil batches failed quality control (QC; **Supplemental Figure S1**) testing. Two testing events (June 2020, and June 2021 assesing near empty bottles returned for the Metabolic Kitchen and destined for discard, did show TOTOX values approaching the upper limit (max =30). The (alpha-lenoleic acid (ALA) content was consistent across all bottles.

Figure S1: Flaxseed Oil: QC Testing for oxidation status (top) and main fatty acid component (bottom, ALA).

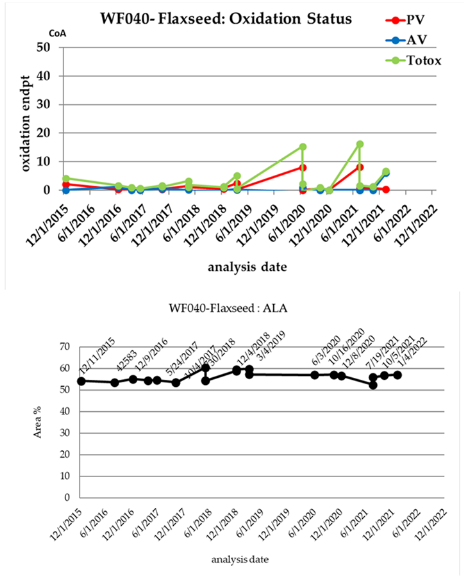

The low-LA safflower oil (**Supplemental Figure S2**) was obtained in 5 shipments (15 5-gallon pails), of which two pails failed QC testing at receipt and another pair failed prior to transfer to the Metabolic Kitchen (indicated in black boxed area) and were therefore discarded. It became necessary to change suppliers in 2020 due to difficulties obtaining product from the supplier. The vetting of new suppliers and due diligence of their product were handled with the rigor demanded by the goal of identifying high quality food-grade products.

The linoleic acid (LA; middle panel) and oleic acid (OA; right panel) profiles of the oil batches and across suppliers were consistent.

Figure S2: Low-LA Safflower Oil: QC Testing for oxidation status (left) and main fatty acid components (LA, middle; OA right).

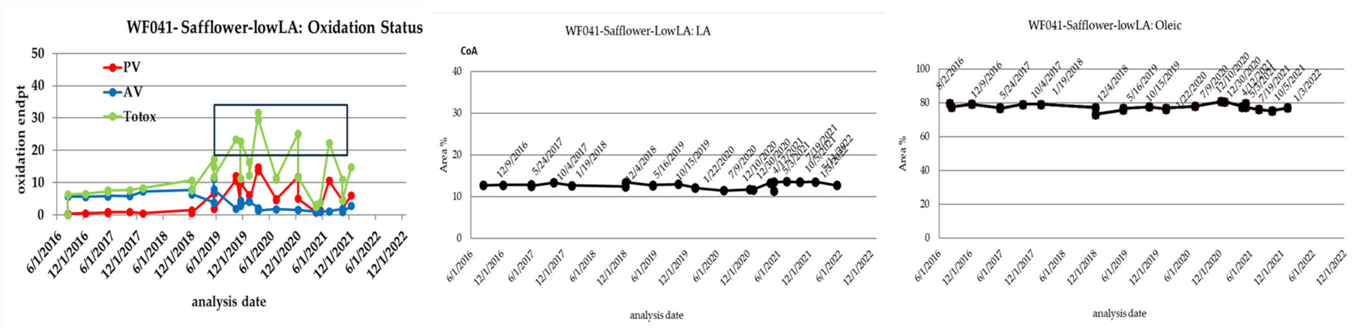

The High-LA safflower oil was obtained in 6 shipments (16 5-gallon pails). The oil product was subjected to QC testing (**Supplemental Figure S3**) over time. Those pail failing QC testing are noted in the black-boxed area in the leftmost panel (Oxidation status). Amoung the oil pails received, 4 pails failed QC testing at receipt, which was attributed to container damage, and were therefore discarded. The prolonged COVID-induced study pause necessitated additional QC testing prior to the study resuming. As a result, a third pail failed this QC testing in 2020 and was dicarded. A fouth pail was discovered to have failed QC testing before its planned transfer to the Metabolic Kitchen. It was discarded. The next pail in line was found to be satisfactoy and taken to the Metabolic Kitchen for use.

It also became necessary to change suppliers in 2019 due to difficulties obtaining product from the initial supplier product discontinuation. The LA (middle panel) and OA (right panel) profiles of the oil batches and across suppliers were consistent.

Figure S3: High-LA Safflower Oil: QC Testing for oxidation status (left) and main fatty acid components (LA, middle; OA, right).

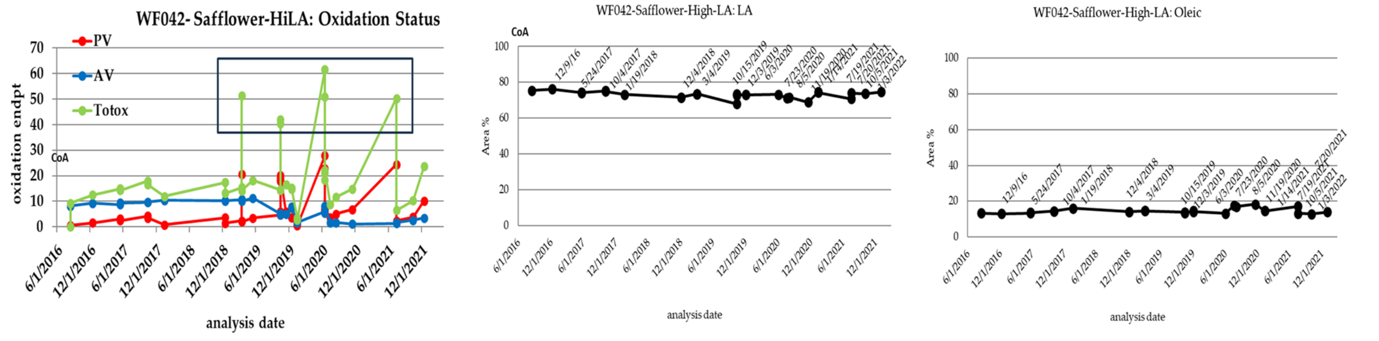

Oxylipin Authentic Standards and Internal Standards

Supplemental Table S2: All authentic oxylipin compounds used to generate standard curves for quantification were obtained from Caymen Chemical (Ann Arbor, MI, USA). Mass transitions used for analyses are shown Deuterated Internal standards were also obtained from Caymen Chemical.

Authentic Oxylipin Compounds

Compound ID	Q1 mass	Q3 mass
11,12 DiHETrE	337.2	167.1
11,12 EET	319.2	167.1
11,12-EpETE	317.2	167.1
11-HETE	319.2	167.1
12(13)-EpOME	295.3	195.2
12,13-DiHOME	313.3	183.2
12-HEPE	317.2	179.1
12-HETE	319.2	179.1
13(S)HOTrE	293.2	195
13,14-dihydro-15-keto PGF2a	353.2	291.1
13-HODE	295.2	195.1
14,15 DiHETrE	337.2	207.1
14,15 EET	319.2	219.1
14,15-DiHETE	335.3	207.2
14,15-EpETE	317.2	207.1
14-HDHA	343.2	205.1
15-deoxy-PGJ2	315.2	271.1
15-HEPE	317.2	219.1
15-HETE	319.2	219.1
15-HETrE	321.2	221.157
15-KETE	317.2	273.2
15-keto-PGE2	349.2	113.1
16,17-EpDPA	343.2	274.1
17,18-DiHETE	335.3	247.2
17,18-EpETE	317.2	259.2
17-HDHA	343.2	245.1
19,20-DiHDPE	361.3	273.2
19,20-EpDPA	343.2	241.1
20-HETE	319.2	289.1
4-HDHA	343.2	101.1

Compound ID	Q1 mass	Q3 mass
5,6 DiHETrE	337.2	145.1
5-HEPE	317.2	115.1
5-HETE	319.2	115.1
5-HETrE	321.2	303.243
5-oxo-ETE	317	203.3
6k-PGF1a	369.2	163.1
6-trans-LTB4	335.3	195.2
8(9)-EpETrE	319.2	155.1
8-HETE	319.2	155.1
8-HETrE	321.2	157.102
9(10)-EpOME	295.2	171.1
9(S)HOTrE	293.3	171.1
9,10 DiHOME	313.3	201.3
9,10-e-DiHO	315.2	297.2
9-HEPE	317.2	167.2
9-HETE	319.3	167.2
9-HODE	295.2	171.1
9-KODE	293.2	185.2
Alpha-12(13)-EpODE	293.2	183.2
LTB4	335.2	195.1
PGD2	351.2	233.1
PGE1	353.1	317.2
PGE2	351.2	175.1
PGE2 and PGD2	351.2	189.1
PGE3	349.3	269.2
PGF2a	353.2	193.1
PGF3a	351.2	193.1
RvD1	375.2	121.1
TXB2	369.2	169.1

Deuterated Interanal Standards

Internal Standards

d11-14,15-DiHETrE	348.4	207.2
d4-12(13)-EpOME	299.2	198.1
d4-6keto-PGF1a	373.3	167.1
d4-9-HODE	299.2	172.1
d4-LTB4	339.2	197.1
d4-PGD2	355.3	275.2
d4-PGF2a	357.3	197.2
d4-TXB2	373.3	173.1
d6-20-HETE	325.3	281.2
d8-12-HETE	327.2	184.1
d8-5-HETE	327.2	116.1

Supplemental Figure S4: Low **Abundance Oxylipins Generated by Zymosan-Stimulation of Whole Blood**

This figure shows an expanded portion of the right side of **Figure 7b** to highlight the lower abundance (>50ng/ml) oxylipins generated during the whole blood stimulation of immune cells by the phagocytic stimulus, zymosan.

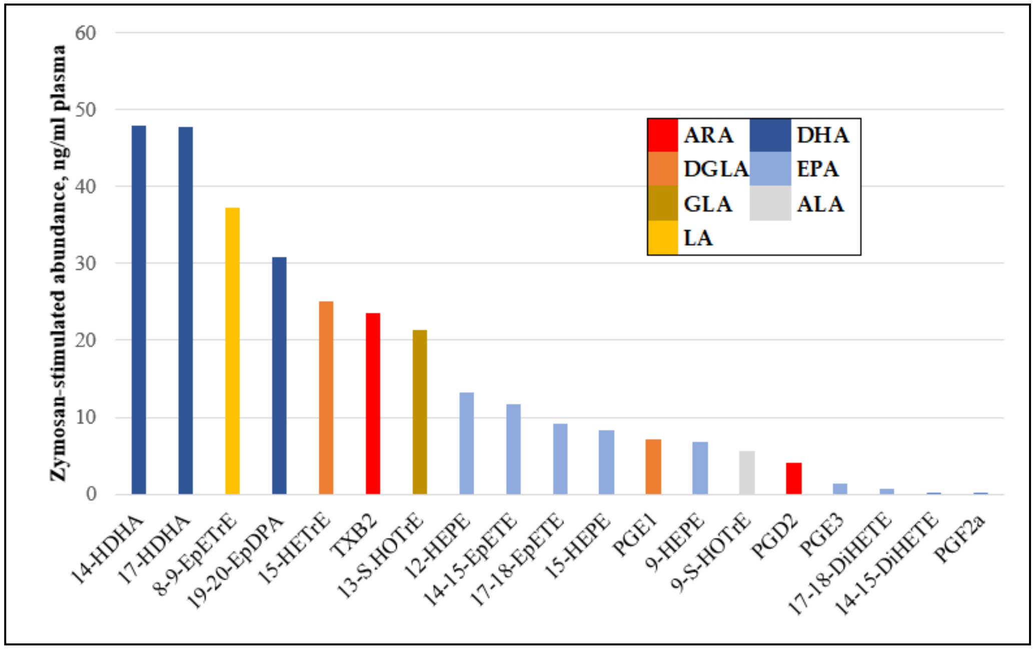

Supplemental Table S3: Baseline Plasma n-6 and n-3 PUFA and HUFA Concentrations in Each Arm. Baseline (Week 0 of intervention) plasma PUFA, HUFA and MUFA concentrations (mg/dl) by study arm and ratios are shown. Data are presented as mean values with the 95% confidence interval. Data from the study arms were analyzed by 2-tailed T-test with the resultant p-value stated.

	Mean	95% CI	*p-value*		Mean	95% CI	*p-value*
n-6 PUFA-HUFA				***n-3 PUFA-HUFA***			
**LA**				**ALA**			
**Low-LA**	82.5	77.4, 87.6	0.232	**Low-LA**	1.421	1.2, 1.6	0.833
**High-LA**	87.2	19.1, 93.5		**High-LA**	1.448	1.2, 1.6	
**GLA**				**EPA**			
**Low-LA**	0.951	0.80, 1.1	0.274	**Low-LA**	1.09	0.9, 1.3	0.938
**High-LA**	1.11	0.9, 1.4		**High-LA**	1.1	0.9, 1.3	
**DGLA**				**DPA**			
**Low-LA**	3.42	3.1, 4.1	0.541	**Low-LA**	0.78	0.7, 0.9	0.668
**High-LA**	3.609	3.1, 4.1		**High-LA**	0.81	0.7, 0.9	
**ARA**				**DHA**			
**Low-LA**	15.3	13.5, 17.2	0.459	**Low-LA**	2.63	2.2, 3.0	0.509
**High-LA**	16.32	14.2, 18.4		**High-LA**	2.87	2.2, 3.5	
n-6 PUFA-HUFA Ratios				***n-3 PUFA-HUFA Ratios***			
**GLA/LA**				**EPA/ALA**			
**Low-LA**	0.011	0.01, 0.013	0.453	**Low-LA**	0.83	0.69, 0.97	0.951
**High-LA**	0.012	0.01, 0.015		**High-LA**	0.84	0.65, 1.02	
**DGLA/GLA**				**DHA/ALA**			
**Low-LA**	4.78	3.8, 5.7	0.441	**Low-LA**	1.96	1.65, 2.27	0.761
**High-LA**	4.29	3.5, 5.1		**High-LA**	2.03	1.65, 2.41	
**ARA/DGLA**				**DHA/EPA**			
**Low-LA**	4.68	4.2, 5.2	0.841	**Low-LA**	2.55	2.22, 2.88	0.6
**High-LA**	4.75	4.2, 5.3		**High-LA**	2.68	2.30, 3.05	

	Mean	95% CI	*p-value*
**Total n-6/n-3**			
**Low-LA**	17.69	16.3, 19.1	0.73
**High-LA**	18.04	16.6, 19.5	
**ARA/EPA**			
**Low-LA**	15.39	13.6, 17.2	0.312
**High-LA**	16.81	14.5, 19.1	
**MUFA**			
**OA**			
**Low-LA**	45.8	42.2, 49.4	0.091
**High-LA**	51.4	45.6, 57.3	

Supplemental Table S4: Mixed-model results for all primary and secondary FA outcomes

Outcomes were log-transformed and adjusted for baseline, age, sex, study week, treatment arm, and the study week × arm interaction, with a random intercept for participant ID. Time was modeled as a factor due to the non-linear appearance of multiple FA trajectories. P-values correspond to the contrast testing for a difference between the two arms at each time point.

Outcome	Fatty Acid	Study week	Contrast	Estimated Ratio of Geometric Means[1]	95% CI	Percent difference	p-value	p_adj
Primary endpoint	LA	12	(High-LA) - (Low-LA)	1.31	(1.22, 1.4)	30.5	2.14E-11	8.55E-11
	EPA	12	(High-LA) - (Low-LA)	0.70	(0.56, 0.87)	-30.2	0.0017	0.0050
	ARA	12	(High-LA) - (Low-LA)	0.95	(0.87, 1.04)	-5.1	0.2483	0.2483
	DHA	12	(High-LA) - (Low-LA)	0.90	(0.8, 1.02)	-9.8	0.1026	0.2052
Primary supportive timepoint	LA	4	(High-LA) - (Low-LA)	1.27	(1.19, 1.36)	27.5	2.14E-10	1.06E-09
	LA	8	(High-LA) - (Low-LA)	1.29	(1.2, 1.38)	28.8	1.22E-10	6.45E-10
	EPA	4	(High-LA) - (Low-LA)	0.68	(0.55, 0.85)	-31.8	0.0007	0.0022
	EPA	8	(High-LA) - (Low-LA)	0.62	(0.5, 0.77)	-38.0	4.38E-05	0.0002
	ARA	4	(High-LA) - (Low-LA)	0.91	(0.84, 1)	-8.6	0.0401	0.0631
	ARA	8	(High-LA) - (Low-LA)	0.95	(0.87, 1.04)	-4.8	0.2734	0.3429
	DHA	4	(High-LA) - (Low-LA)	0.88	(0.78, 0.99)	-12.0	0.0340	0.0559
	DHA	8	(High-LA) - (Low-LA)	0.89	(0.78, 1)	-11.3	0.0563	0.0850
Secondary endpoint	ADA	4	(High-LA) - (Low-LA)	1.02	(0.9, 1.15)	1.6	0.7964	0.8419
	ADA	8	(High-LA) - (Low-LA)	0.99	(0.87, 1.12)	-1.1	0.8664	0.8782
	ADA	12	(High-LA) - (Low-LA)	0.99	(0.87, 1.12)	-1.2	0.8476	0.8711
	ALA	4	(High-LA) - (Low-LA)	0.85	(0.72, 1.01)	-14.8	0.0678	0.1003
	ALA	8	(High-LA) - (Low-LA)	0.81	(0.67, 0.97)	-19.5	0.0197	0.0356
	ALA	12	(High-LA) - (Low-LA)	0.82	(0.69, 0.99)	-17.7	0.0361	0.0581

DGLA	4	(High-LA) - (Low-LA)	0.87	(0.77, 0.98)	-13.3	0.0182	0.0336
DGLA	8	(High-LA) - (Low-LA)	0.82	(0.73, 0.93)	-17.9	0.0018	0.0052
DGLA	12	(High-LA) - (Low-LA)	0.83	(0.73, 0.94)	-17.0	0.0032	0.0088
DPA	4	(High-LA) - (Low-LA)	0.84	(0.73, 0.96)	-16.2	0.0112	0.0224
DPA	8	(High-LA) - (Low-LA)	0.85	(0.74, 0.98)	-15.2	0.0219	0.0377
DPA	12	(High-LA) - (Low-LA)	0.88	(0.76, 1.01)	-12.0	0.0750	0.1067
ETA	4	(High-LA) - (Low-LA)	0.83	(0.66, 1.03)	-17.5	0.0928	0.1272
ETA	8	(High-LA) - (Low-LA)	0.65	(0.51, 0.82)	-34.8	0.0005	0.0018
ETA	12	(High-LA) - (Low-LA)	0.68	(0.54, 0.86)	-31.9	0.0017	0.0052
GLA	4	(High-LA) - (Low-LA)	1.03	(0.77, 1.37)	3.1	0.8331	0.8683
GLA	8	(High-LA) - (Low-LA)	0.86	(0.64, 1.15)	-14.3	0.2986	0.3623
GLA	12	(High-LA) - (Low-LA)	0.98	(0.73, 1.31)	-2.1	0.8886	0.8886
Oleic	4	(High-LA) - (Low-LA)	0.59	(0.53, 0.65)	-41.4	3.90E-17	9.62E-16
Oleic	8	(High-LA) - (Low-LA)	0.51	(0.46, 0.56)	-49.2	1.77E-22	1.31E-20
Oleic	12	(High-LA) - (Low-LA)	0.52	(0.46, 0.57)	-48.4	9.61E-22	3.55E-20
Palmitic	4	(High-LA) - (Low-LA)	0.93	(0.86, 1.01)	-6.8	0.0837	0.1168
Palmitic	8	(High-LA) - (Low-LA)	0.87	(0.8, 0.95)	-13.0	0.0013	0.0043
Palmitic	12	(High-LA) - (Low-LA)	0.88	(0.81, 0.95)	-12.4	0.0022	0.0062
SDA	4	(High-LA) - (Low-LA)	1.07	(0.89, 1.28)	6.6	0.4863	0.5530
SDA	8	(High-LA) - (Low-LA)	0.92	(0.76, 1.12)	-7.6	0.4163	0.4814
SDA	12	(High-LA) - (Low-LA)	0.94	(0.77, 1.13)	-6.4	0.4933	0.5530
Stearic	4	(High-LA) - (Low-LA)	1.07	(0.99, 1.14)	6.5	0.0720	0.1045
Stearic	8	(High-LA) - (Low-LA)	1.01	(0.94, 1.08)	1.0	0.7862	0.8419
Stearic	12	(High-LA) - (Low-LA)	1.01	(0.94, 1.09)	1.4	0.7002	0.7619

[1]Ratios greater than 1 indicate higher estimated concentration in the 10% High-LA arm relative to the 2.5% Low-LA arm.

Supplemental Table S5: Mixed-model results for the ratio outcomes (secondary outcomes).

Mixed-model results for the ratio outcomes (secondary outcomes). Ratios were log-transformed and adjusted for baseline, age, sex, study week, treatment arm, and the study week × arm interaction, with a random intercept for participant ID. Time was modeled as a factor due to the non-linear appearance of multiple FA trajectories. P-values correspond to the contrast testing for a difference between the two arms at each time point.

Outcome	Ratio	Study week	Contrast	Estimated Ratio of Geometric Means[1]	95% CI	Percent difference	p-value	p_adj
Secondary endpoint	ARA/EPA	4	(High-LA) - (Low-LA)	1.36	(1.1, 1.69)	36.18	0.005	0.013
Secondary endpoint	ARA/EPA	8	(High-LA) - (Low-LA)	1.57	(1.26, 1.95)	56.55	1.04E-04	4.05E-04
Secondary endpoint	ARA/EPA	12	(High-LA) - (Low-LA)	1.39	(1.12, 1.73)	38.80	3.74E-03	0.01
Secondary endpoint	ARA/DHA	4	(High-LA) - (Low-LA)	1.04	(0.95, 1.14)	4.28	0.35	0.42
Secondary endpoint	ARA/DHA	8	(High-LA) - (Low-LA)	1.08	(0.98, 1.18)	7.52	0.13	0.17
Secondary endpoint	ARA/DHA	12	(High-LA) - (Low-LA)	1.05	(0.96, 1.16)	5.45	0.26	0.33
Secondary endpoint	ARA/DGLA	4	(High-LA) - (Low-LA)	1.06	(0.95, 1.2)	6.46	0.30	0.36
Secondary endpoint	ARA/DGLA	8	(High-LA) - (Low-LA)	1.17	(1.04, 1.33)	17.38	0.011	0.022
Secondary endpoint	ARA/DGLA	12	(High-LA) - (Low-LA)	1.16	(1.02, 1.31)	15.72	0.020	0.036
Secondary endpoint	n6/n3 all PUFA+HUFA	4	(High-LA) - (Low-LA)	1.43	(1.32, 1.56)	43.47	9.10E-14	9.62E-13
Secondary endpoint	n6/n3 all PUFA+HUFA	8	(High-LA) - (Low-LA)	1.49	(1.37, 1.63)	49.19	3.61E-15	6.68E-14
Secondary endpoint	n6/n3 all PUFA+HUFA	12	(High-LA) - (Low-LA)	1.46	(1.34, 1.59)	45.60	7.20E-14	8.88E-13
Secondary endpoint	5-HETE/5-HEPE	4	(High-LA) - (Low-LA)	1.56	(1.13, 2.14)	55.57	0.007	0.016
Secondary endpoint	5-HETE/5-HEPE	8	(High-LA) - (Low-LA)	1.49	(1.09, 2.06)	49.40	0.014	0.027
Secondary endpoint	5-HETE/5-HEPE	12	(High-LA) - (Low-LA)	1.53	(1.11, 2.1)	52.74	0.010	0.021
Secondary endpoint	5-HETE/5-HETrE	4	(High-LA) - (Low-LA)	1.82	(1.49, 2.22)	82.36	5.50E-08	2.54E-07
Secondary endpoint	5-HETE/5-HETrE	8	(High-LA) - (Low-LA)	2.12	(1.73, 2.59)	111.71	9.11E-11	5.62E-10
Secondary endpoint	5-HETE/5-HETrE	12	(High-LA) - (Low-LA)	2.45	(2, 2.99)	144.80	1.43E-13	1.18E-12

[1]Ratios greater than 1 indicate higher estimated concentration in the 10% High-LA arm relative to the 2.5% Low-LA arm.
